# A multicenter, retrospective, observational study of the clinical outcomes and risk factors for relapse of ulcerative colitis at 1 year after leukocytapheresis

**DOI:** 10.1007/s00535-017-1356-8

**Published:** 2017-06-08

**Authors:** Taku Kobayashi, Katsuyoshi Matsuoka, Yoko Yokoyama, Takashi Nakamura, Tomoko Ino, Toyoko Numata, Hiroshi Shibata, Hirofumi Aoki, Yoshihiro Matsuno, Toshifumi Hibi

**Affiliations:** 1 Center for Advanced IBD Research and Treatment, Kitasato Institute Hospital, Kitasato University, 5-9-1 Shirokane, Minato-ku, Tokyo, 108-8642 Japan; 2 Department of Gastroenterology and Hepatology, Tokyo Medical and Dental University, Tokyo, Japan; 3 Division of Inflammatory Bowel Disease, Department of Internal Medicine, Hyogo College of Medicine, Hyogo, Japan; 4 Blood Purification Business Division, Asahi Kasei Medical Co. Ltd., Tokyo, Japan

**Keywords:** Ulcerative colitis, Leukocytapheresis, Long-term outcome

## Abstract

**Background:**

Extracorporeal leukocytapheresis (LCAP) is effective for inducing remission of ulcerative colitis (UC). This retrospective observational study aimed to evaluate the clinical outcome at 1 year and identify risk factors for relapse of UC after LCAP.

**Methods:**

Patients with active UC treated with LCAP between 2010 and 2012 were enrolled from 54 medical facilities in Japan. Clinical data evaluated at 1 year after the last LCAP session included the incidence of relapse, 1-year cumulative relapse-free rate, risk factors for relapse, and history of re-induction treatment following relapse. Relapse was defined by the addition of treatment to induce remission. The primary endpoint was the 1-year cumulative relapse-free rate. Secondary endpoints were risk factors for relapse and outcomes of re-induction treatment after relapse.

**Results:**

For 314 patients, the 1-year cumulative relapse-free rate was 63.6%. Following LCAP, a Lichtiger clinical activity index (CAI) of 3 or 4 and high leukocyte count (cut-off value: 7790/mm^3^) were associated with a greater risk of relapse. Intensive LCAP (≥4 sessions within the first 2 weeks) was associated with favorable long-term outcomes in corticosteroid-refractory patients. The response rate was 85.1% among 30 patients who required re-treatment with LCAP.

**Conclusions:**

The majority of patients (>60%) with UC treated with LCAP achieved clinical remission within 1 year and remained relapse-free. A higher Lichtiger CAI and leukocyte count following LCAP were risk factors for relapse. Re-induction therapy with LCAP was effective for relapse of UC.

**Electronic supplementary material:**

The online version of this article (doi:10.1007/s00535-017-1356-8) contains supplementary material, which is available to authorized users.

## Introduction

Ulcerative colitis (UC) is an inflammatory bowel disease with an unclear etiology that mainly affects the mucosa of the colon, causing erosion and ulceration. Lesions in UC continuously affect the mucosa proximally from the rectum to the entire colon and are characterized by erosions and/or ulcerations. Typical clinical symptoms of UC include repeated and persistent bloody stools and abdominal pain [1]. Once UC develops, it cannot be completely cured and cycles of relapse and remission are frequently repeated during the course of the disease. Therefore, it is essential not only to establish an appropriate treatment to induce remission but also to predict the treatment outcome to effectively maintain remission.

Oral and topical 5-amino salicylic acid (5-ASA) is the standard treatment to induce remission of active UC [2]; however, for patients unresponsive to these medications, oral administration of corticosteroids is the treatment of choice [3]. Furthermore, treatment with cytapheresis (CAP), anti-TNFα [4], or tacrolimus (TAC) [5] can be used for corticosteroid-resistant and/or -dependent patients who fail to achieve clinical remission. Once remission is achieved, long-term use of oral 5-ASA is the standard treatment to maintain remission. However, it is necessary to avoid long-term use of corticosteroid therapy because of the risk of various side effects, such as moon face, infectious disease, and osteoporosis [6], and the lack of efficacy to maintain remission [7]. Therefore, maintenance treatment with thiopurines is recommended for corticosteroid-dependent patients to withdraw corticosteroid therapy. Moreover, biologics can be continued to maintain remission when use is effective to induce remission [4, 8].

Leukocytapheresis (LCAP), which is one of the two forms of CAP, was performed using a Cellsorba E column (Asahi Kasei Medial Co., Ltd., Tokyo, Japan) filled with nonwoven polyester fiber. LCAP, which was approved for use in Japan in 2001, exerts anti-inflammatory effects by removing activated leukocytes and platelets from the peripheral blood through extracorporeal circulation [9–12].

An open-label, multicenter, randomized, controlled trial reported that LCAP was effective in 74% of 39 corticosteroid-resistant patients [13]. Furthermore, a double-blind, controlled trial using a sham column demonstrated that LCAP was effective in 80% of ten patients with corticosteroid-resistant UC and superior to the use of a sham column [14]. Thereafter, a large-scale, post-marketing, observational study conducted by our group between 2010 and 2012 found that the rates of clinical remission and mucosal healing with LCAP were 68.9% (*n* = 429 of 623) and 62.5% (*n* = 145 of 232), respectively. That study also demonstrated significantly higher clinical remission and mucosal healing rates with the use of intensive LCAP (≥4 sessions within the first 2 weeks), rather than weekly LCAP [15].

Although there are many reports of the efficacy of LCAP as a remission-induction therapy, data of long-term outcomes remain insufficient. Therefore, the aims of this multicenter, retrospective, observational study were to evaluate the incidence of relapse within 1 year after LCAP, assess the long-term treatment outcomes, and identify risk factors for relapse. Moreover, outcomes of re-induction treatment in patients who had relapsed during the observation period were also assessed.

## Methods

### Study design

A previous post-marketing study, conducted between 2010 and 2012, included 623 patients to evaluate the efficacy of LCAP after excluding those treated with LCAP in combination with infliximab (IFX), TAC, or cyclosporine. Of these patients, those who achieved clinical remission at 2 weeks after the last LCAP session were eligible for the current multicenter, retrospective, observational study. Clinical remission was defined as a Lichtiger clinical activity index (CAI) of ≤4 [16].

**Fig. 1:**
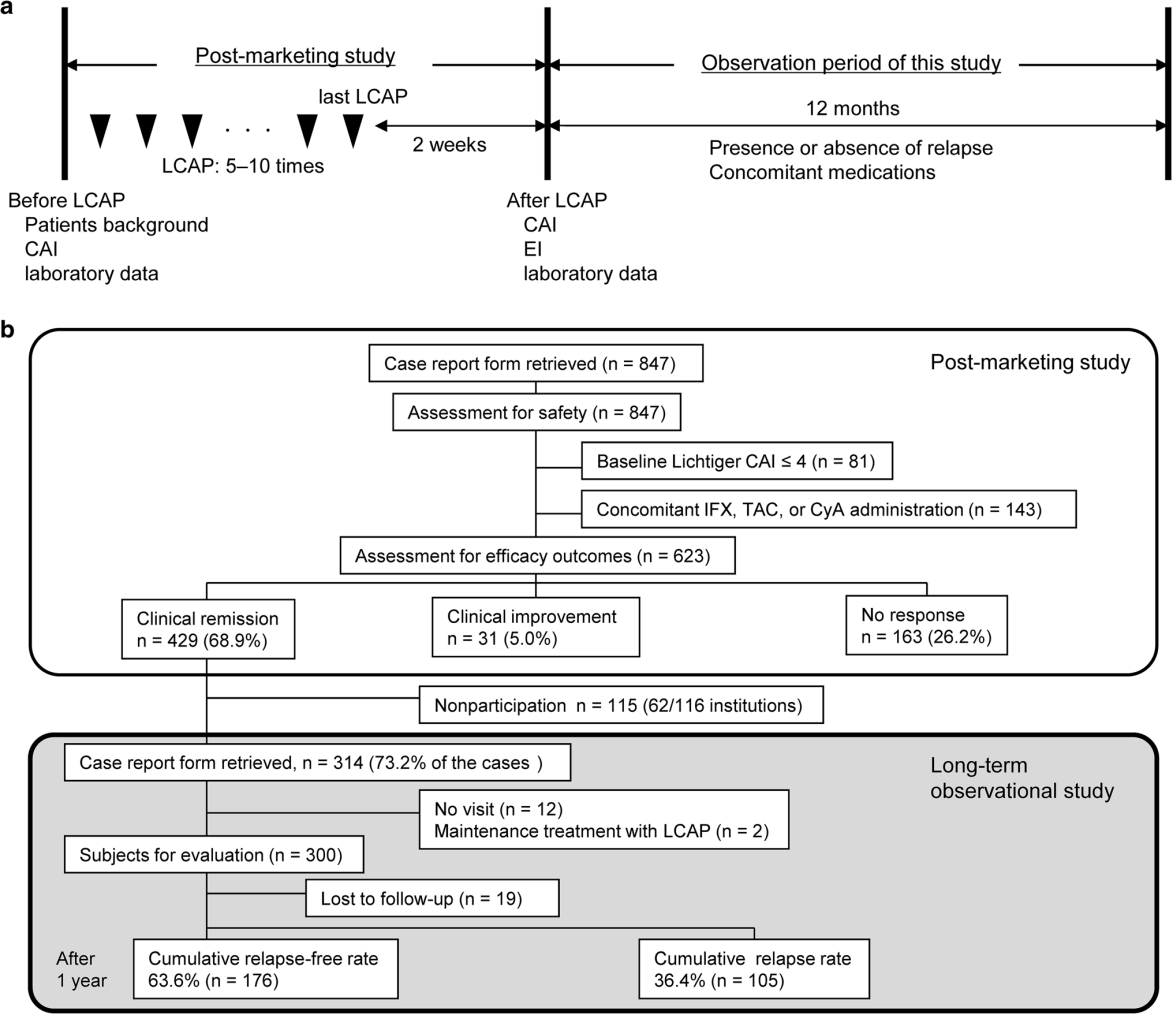
Study outline. **a** Timelines of the previous postmarketing study and the present long-term observational study. **b** Summaries of the previous postmarketing study and the present long-term observational study. *CAI* clinical activity index, *CyA* cyclosporine, *EI* endoscopic index, *IFX* infliximab, *LCAP* leukocytapheresis, *TAC* tacrolimus

Of 116 institutions that participated in the previous postmarketing study, 54 (46.6%) participated in the current study. A total of 5–10 sessions of LCAP (mean 9.1 ± 1.9 sessions) were performed as an induction treatment. Intensive LCAP was defined as ≥4 sessions within the first 2 weeks. Clinical activity and laboratory data were evaluated at baseline (before LCAP) and 2 weeks after the last LCAP session (after LCAP), and then the patients were followed for 1 year after LCAP (Fig. 1a). The presence or absence of relapse, date of relapse, and severity of relapse during the observation period were evaluated. Furthermore, concomitant medications, re-induction treatment, and outcomes of re-induction treatment of patients who relapsed during the observation period were evaluated. The primary endpoint was the 1-year cumulative relapse-free rate. Secondary endpoints were risk factors for relapse and outcomes of re-induction treatment after relapse.

The severity of relapse was evaluated by partial Mayo scores, which excluded the endoscopic subscores from the full Mayo scores [2]. Outcomes of re-induction treatment in patients who relapsed were classified as “remission”, “improvement”, or “no response” based on evaluations conducted by attending physicians.

The information collected in the previous postmarketing study provided age, weight, sex, disease duration, CAI, response to corticosteroids and laboratory data before LCAP; and CAI, endoscopic index (EI) and laboratory data after LCAP; and concomitant medications during LCAP treatment and LCAP treatment status.

Failure to respond to systemic corticosteroids at a dose of 1–1.5 mg/kg/day for 1 week was considered as corticosteroid resistant. Corticosteroid-dependent was defined when withdrawal of corticosteroids had failed. Corticosteroid-resistant and corticosteroid-dependent cases were considered as refractory. Endoscopic severity was graded according to the EI of the disease activity index [17], and the EI after LCAP was collected when available. Use of other treatments at any time point during LCAP treatment and/or the observation period was considered as “concomitant medication”.

The study protocol was approved by the institutional review boards of all 54 participating institutions.

### Definition of relapse

Relapse was defined by the addition of the following remission-induction treatments: oral and intravenous injection of corticosteroids, LCAP, granulocyte and monocyte adsorptive therapy (GMA), IFX, adalimumab (ADA), TAC, and surgery. Moreover, an increase in the corticosteroid dose was considered as relapse.

### Statistical analysis

All statistical analyses were conducted using SAS ver. 9.3 software (SAS Institute, Cary, NC, USA).

Clinical relapse, as defined earlier, was considered as an event occurrence and patients who remained relapse-free were followed-up at the end of the observation period. The 1-year cumulative relapse-free rate after LCAP was assessed using the Kaplan–Meier method.

Risk factors for relapse among background characteristics were identified by univariate analysis using the log-rank test. Furthermore, continuous values of laboratory data were evaluated with the Cox proportional hazards model. Then, the Cox proportional hazards model was utilized to analyze factors that showed statistical significance by univariate analysis. A variable selection during the multivariate analysis was conducted according to the step-wise method.

Cut-off values under the receiver-operating characteristic (ROC) curve were calculated based on the maximum value of the Youden index. A probability (*p*) value of <0.05 was considered statistically significant, and 95% confidence intervals were calculated.

## Results

### Patient dispositions and backgrounds

For 429 patients who achieved clinical remission in the previous postmarketing study, case report forms were collected from 314 (73.2%) (Fig. 1b). For these 314 patients, 12 who did not return to the hospital for follow-up and two who continued LCAP as a maintenance therapy were excluded. The remaining 300 patients were enrolled in this study. A total of 281 patients completed the 1-year follow-up.

The following information is included in Table 1: (1) patient background and laboratory data before LCAP; and (2) CAI, EI, and laboratory data after LCAP (beginning of the observation period) and Table 2: concomitant medications and LCAP treatment status. The average ± standard deviation baseline CAI before LCAP was 10.2 ± 3.2 and 87.0% of patients had moderate-to-severe active UC. Moreover, 64.8% of patients (*n* = 193) with UC were corticosteroid-refractory. The mean CAI after LCAP was 1.8 ± 1.3. More than 90% of patients received 5-ASA during LCAP and the observation period, 33.7% received thiopurine maintenance therapy during LCAP, and 43.3% received thiopurine maintenance therapy during the observation period. At the beginning of the observation period, 54.3% of the patients were on corticosteroids. Intensive LCAP was performed in 69.8% of the patients.

**Table 1:** Patient backgrounds

Item	No. of patients	Mean ± SD (minimum–maximum), %
Before LCAP		
Age, years	300	41.2 ± 16.2 (14.0–85.0)
Weight, kg	276	57.3 ± 11.2 (23.0–88.0)
Sex		
Male	184	61.3%
Female	116	38.7%
Disease duration, years	293	7.3 ± 7.7 (0–42.8)
Lichtiger CAI	300	10.2 ± 3.2 (5.0–21.0)
Clinical activity		
Mild, CAI = 5–6	39	13.0%
Moderate, CAI = 7–11	159	53.0%
Severe, CAI ≥ 12	102	34.0%
Disease extent		
Total	171	57.2%
Left sided	110	36.8%
Others	18	6.0%
Response to corticosteroid		
Resistant	86	28.9%
Dependent	107	35.9%
Non-refractory	105	35.2%
Previous use of corticosteroid	226	75.6%
Laboratory data		
Leukocyte count, /mm^3^	290	8766 ± 3398 (2500–24700)
Erythrocyte count, ×10^4^/mm^3^	290	439.0 ± 62.1 (184.0–581.0)
Platelet count, ×10^4^/mm^3^	290	32.8 ± 11.3 (9.3–107.9)
Hemoglobin level, g/dl	290	12.5 ± 2.1 (5.4–17.4)
CRP level, mg/dl	290	2.3 ± 4.2 (0.0–29.8)
Erythrocyte sedimentation rate, mm/h	155	36.0 ± 30.3 (2.0–135.0]
After LCAP		
Lichtiger CAI	300	1.8 ± 1.3 (0–4.0)
EI	106	0.9 ± 0.8 (0–2)
Laboratory data		
Leukocyte count, /mm^3^	265	7263.0 ± 2716.0 (2600–18300)
Erythrocyte count, ×10^4^/mm^3^	264	426.5 ± 58.5 (261.0–553.0)
Platelet count, ×10^4^/mm^3^	264	28.9 ± 9.0 (9.3–66.4)
Hemoglobin level, g/dl	264	12.3 ± 2.1 (6.9–25.4)
CRP level, mg/dl	263	0.3 ± 0.5 (0.0–3.5)
Erythrocyte sedimentation rate, mm/h	138	18.6 ± 19.5 (2.0–130.0)

*CAI* clinical activity index, *CRP* C-reactive protein, *EI* endoscopic index, *LCAP* leukocytapheresis

**Table 2:** Concomitant medications and LCAP treatment status

Item	No. of patients	%
Concomitant medications		
During LCAP		
5-ASA	286	95.3
Thiopurine	101	33.7
Corticosteroid	183	61.0
Beginning of the observation period		
Corticosteroid	163	54.3
During the observation period		
5-ASA	280	93.3
Thiopurine	130	43.3
LCAP treatment status		
Number of sessions		
5–7	46	15.8
8–9	17	5.8
10	228	78.4
Frequency of LCAP		
Weekly	88	30.2
Intensive	203	69.8

*5-ASA* 5-aminosalicylic acid, *LCAP* leukocytapheresis

### 1-Year cumulative relapse-free rate

**Fig. 2:**
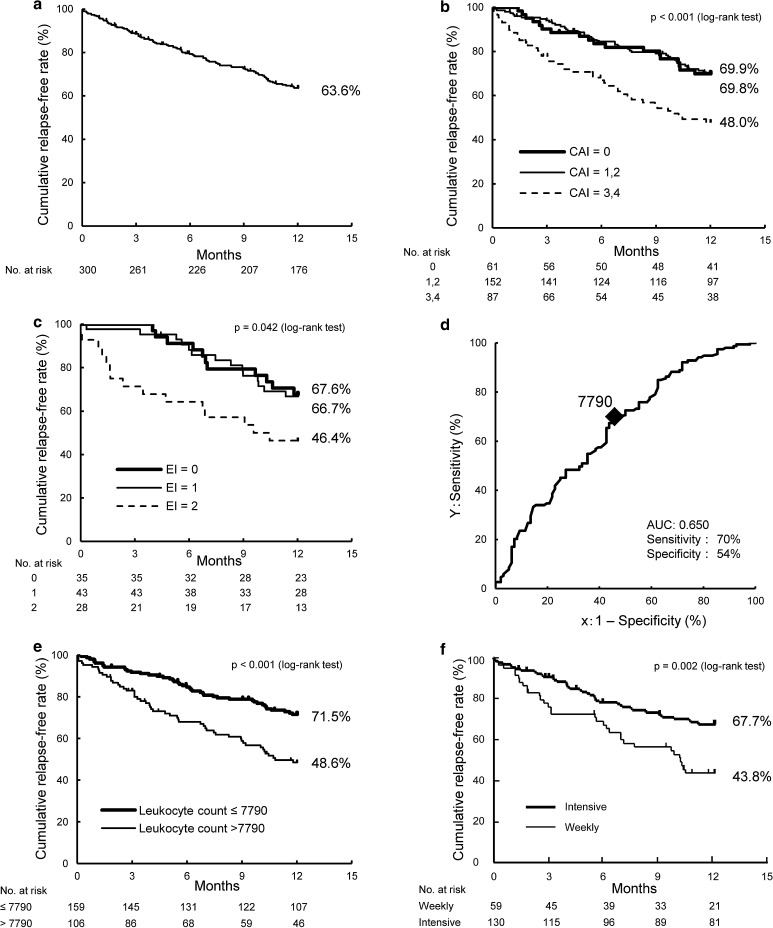
Kaplan–Meier curves of the cumulative relapse-free rate and cut-off value of the leukocyte count after LCAP for the risk of relapse. **a** The overall cumulative relapse-free rate (*n* = 300). **b** Comparison of the cumulative relapse-free rates by CAI score after LCAP (*n* = 300). **c** Comparison of the cumulative relapse-free rates by EI score after LCAP (*n* = 106). **d** ROC curve for the cut-off value of the leukocyte count after LCAP for the risk of relapse (*n* = 249). **e** Comparison of the cumulative relapse-free rates of a leukocyte count of ≤7790 vs. >7790/mm^3^ (*n* = 265). **f** Comparison of the cumulative relapse-free rates of intensive LCAP vs. weekly LCAP in corticosteroid-refractory patients (*n* = 189). *CAI* clinical activity index, *EI* endoscopic index, *LCAP* leukocytapheresis, *ROC* receiver-operating characteristics

The cumulative relapse-free rate was analyzed by the Kaplan–Meier method. As shown in Fig. 2a, the 1-year, cumulative, relapse-free rate was 63.6% (*n* = 176). Furthermore, the 3- and 6-month remission rates were 88.6 and 79.3%, respectively.

Relapse occurred at an average of 163.8 ± 108.4 days. For the 176 patients who remained remission free for 1 year, 100 (56.8%) received simultaneous corticosteroid therapy during LCAP and 87.0% (87 of 100) were successfully withdrawn from corticosteroid therapy during the observation period. Five patients eventually required colectomy.

### Risk factors for relapse

Patient background and laboratory data before LCAP treatment and CAI, EI, and laboratory data after LCAP as well as concomitant medications and LCAP treatment status were analyzed to identify risk factors for relapse after LCAP treatment. Univariate analysis demonstrated that refractory to corticosteroid therapy, CAI = 3 or 4 after LCAP (Fig. 2b), EI = 2 after LCAP (Fig. 2c), higher leukocyte count after LCAP, and concomitant use of corticosteroid therapy after LCAP were risk factors for relapse (Tables 3, S1).

**Table 3:** Risk factors for relapse (univariate analysis)

Risk factor	No. of patients	1-Year cumulative relapse-free rate (%)	*p* value
Response to corticosteroid			
Refractory	193	60.5	0.049^a^
Non-refractory	105	70.5	
CAI after LCAP			
CAI = 0	61	69.9	<0.001^a^
CAI = 1, 2	152	69.8	
CAI = 3, 4	87	48.0	
EI after LCAP			
EI = 0	35	67.6	0.042^a^
EI = 1	43	66.7	
EI = 2	28	46.4	
Leukocyte count after LCAP			
Continuous value (1000/mm^3^ U)	265	–	<0.001^b^
Concomitant corticosteroid after LCAP			
Yes	163	58.6	0.034^a^
No	137	69.6	

*CAI* clinical activity index, *EI* endoscopic index, *LCAP* leukocytapheresis

^a^Calculated using the log-lank test

^b^Calculated using the Cox proportional hazards model

Multivariate analysis identified CAI = 3 or 4 after LCAP and higher leukocyte count after LCAP as independent risk factors for relapse (Table 4). A higher leukocyte count after LCAP was a significant risk factor for relapse, regardless of concomitant use of corticosteroids or thiopurines.

**Table 4:** Risk factors for relapse (multivariate analysis)

Risk factor	HR	95% CI	*p* value
CAI after LCAP: CAI = 3, 4	2.09	1.06–4.13	0.033^a^
Leukocyte count after LCAP: continuous value (1000/mm^3^ U)	1.14	1.02–1.26	0.017^a^

*CAI* clinical activity index, *LCAP* leukocytapheresis

^a^Calculated using the Cox proportional hazards model

Next, a cut-off value of the leukocyte count after LCAP for the risk of relapse was determined by ROC analysis. A cut-off value of the leukocyte count of 7790/mm^3^ resulted in a sensitivity of 70% and specificity of 54% (Fig. 2d). Kaplan–Meier curves of cumulative relapse-free survival stratified by this cut-off value were constructed, which showed that the cumulative relapse-free rates for cut-off values of ≤7790/mm^3^ (*n* = 159) and >7790/mm^3^ (*n* = 106) were 71.5 and 48.6%, respectively (*p* < 0.001; Fig. 2e). The cumulative relapse-free rate associated with a leukocyte count of ≤7790/mm^3^ was significantly higher than that associated with a count of >7790/mm^3^ regardless of concomitant use of corticosteroids or thiopurines.

When limited to corticosteroid-refractory patients (*n* = 193), the relapse-free rate after intensive LCAP was significantly higher than that after weekly LCAP (*p* = 0.002, Fig. 2f). This result remained significant in multivariate analysis. On the other hand, there was no statistical difference between intensive and weekly LCAP in corticosteroid-non-refractory patients.

### Relapse rates according to 5-ASA dosage and use of concomitant thiopurine maintenance therapy during the observation period

Next, the effects of medication on remission maintenance after LCAP therapy were analyzed. A total of 184 patients (61.3%) received high-dose 5-ASA (high-dose group: time-dependent-release mesalazine at 4000 mg/day, pH-dependent release mesalazine at 3600 mg/day) during the observation period and 54 (18.0%) received low-dose 5-ASA (low-dose group; doses less than the above). There was no significant difference in cumulative relapse-free rates between the high- and low-dose groups (*p* = 0.610).

A total of 116 patients (38.7%) received thiopurine maintenance therapy during LCAP treatment or the observation period, while 165 (55.0%) did not. Again, there was no significant difference in the cumulative relapse-free rate between those who did and did not receive concomitant thiopurine maintenance therapy (*p* = 0.664). However, when limited to corticosteroid-non-refractory patients (*n* = 105), the cumulative relapse-free rate was significantly higher in patients who received concomitant thiopurine maintenance therapy than in those who did not (93.1 vs. 61.6%, respectively, *p* = 0.003).

### Re-induction treatment

There were 105 patients who achieved remission with LCAP, but later relapsed during the 1-year follow-up period. The mean partial Mayo score at the time of relapse was 5.7 ± 1.7. For these patients, re-induction treatment included corticosteroid monotherapy (*n* = 38; 36.2%), LCAP (*n* = 30; 28.6%), TAC (*n* = 14; 13.3%), IFX (*n* = 10; 9.5%), GMA (*n* = 8; 7.6%), ADA (*n* = 2; 1.9%), and others (*n* = 3; 2.9%).

**Fig. 3:**
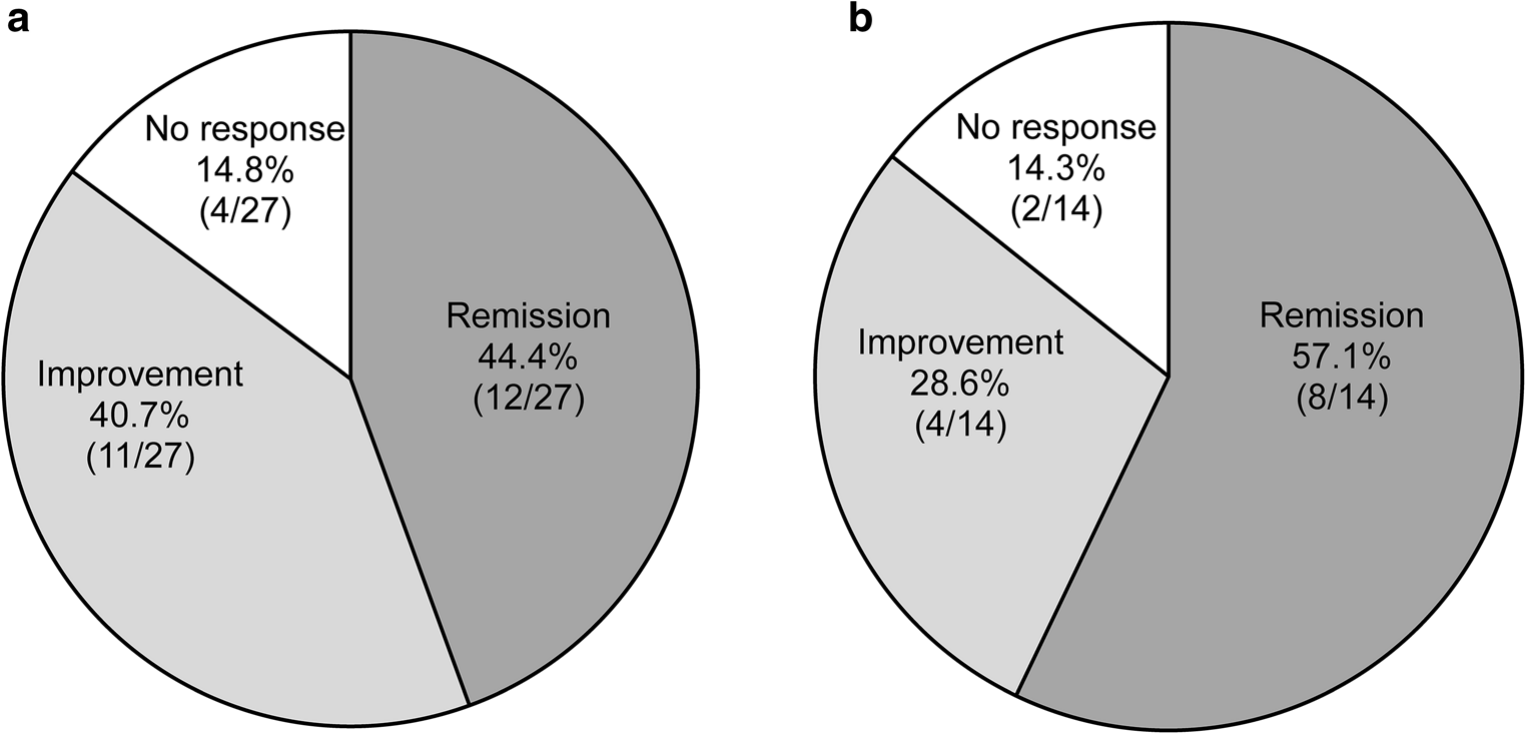
Outcomes after LCAP re-treatment. **a** Re-induction treatment with LCAP. Three patients with unknown outcomes were excluded. **b** Re-induction treatment with LCAP monotherapy. One patient with unknown outcome was excluded. *LCAP* leukocytapheresis

Of 30 patients who used LCAP as part of a re-induction treatment, 12 (44.4%) achieved remission, disease improved in 11 (40.7%), and four (14.8%) did not respond to treatment (Fig. 3a). Moreover, of 15 patients who were re-treated with LCAP alone, eight (57.1%) achieved remission, disease improved in four (28.6%), and two (14.3%) did not respond to treatment (Fig. 3b).

## Discussion

Here, the 1-year cumulative relapse-free rate and risk factors for relapse in a large-scale retrospective study of patients with UC who achieved remission with LCAP are reported. It is worth noting that this study is the first to evaluate the 1-year outcome of LCAP in a large patient cohort to reflect actual clinical practice [18]. The data derived from this study will enable clinicians to better tailor treatment plans for patients with UC.

Faubion et al. [19] reported that corticosteroid treatment resulted in clinical improvement in 49% of patients with UC at 1 year after treatment, and all were successfully withdrawn from corticosteroid therapy. In addition to LCAP, GMA using Adacolum (JIMRO, Takasaki, Japan), which removes granulocytes and monocytes, has been used as a CAP therapy for moderate-to-severe active UC. A clinical trial of GMA in patients with moderate-to-severe UC indicated that the remission maintenance rate was 38.8% (52 of 134) at 12 months after induction of remission [20]. Furthermore, the relapse-free survival rate of 36 patients at 12 months after TAC therapy followed by maintenance treatment with azathioprine was 37% [21]. In contrast, there are other treatment options available for maintenance of remission, which include IFX and ADA. Symptomatic remission rates at week 54 in patients who achieved mucosal healing at week 8 by IFX were reported to be 73% (41 of 56) and 47% (43 of 91) in patients with Mayo endoscopic subscores of 0 and 1, respectively [22]. Although the data of the present study cannot be directly compared with the data of previous studies because of different definitions of relapse and patient backgrounds, the results of the present study showed that, once patients achieved remission with LCAP, approximately two thirds remained relapse-free for 1 year.

Long-term use of corticosteroids should be avoided because of the various side effects caused by such treatment. About 90% of patients who successfully achieved remission with LCAP and remained relapse-free for 1 year were able to discontinue corticosteroid therapy. Hence, LCAP seems useful to withdraw patients from corticosteroid therapy after induction of remission.

Risk factors for relapse during the observation period were a higher CAI and leukocyte count after LCAP treatment. These results suggest that patients who remained mildly inflamed after achieving remission were more likely to relapse since a higher CAI and leukocyte count may reflect residual inflammation. Although leukocyte count might be influenced by the concomitant use of corticosteroids or thiopurines, a higher leukocyte count after LCAP remained a significant risk factor for relapse both with and without the use of concomitant corticosteroids or thiopurines. These results indicate that a higher leukocyte count after LCAP is a significant risk factor for relapse, regardless of the concomitant use of corticosteroids or thiopurines.

Moreover, the EI after LCAP was also identified as a risk factor for relapse by univariate analysis, although this index was not significant by multivariate analysis. It has been reported that mucosal healing after induction treatment is associated with favorable outcomes [23]; therefore, it is also possible that endoscopic improvement by LCAP may be predictive of a better clinical course. Overall, “deep remission” after LCAP, as defined by the laboratory data and endoscopic findings, seems important for long-term maintenance of remission.

The results of this study showed that a leukocyte count cut-off value of 7790/mm^3^ after LCAP was a predictor of relapse. In daily clinical practice, CAI, EI, and leukocyte count after LCAP could be used as predictors of relapse, and when these factors predict relapse, it is important to follow-up the patients with caution and optimize the maintenance strategy.

On the other hand, when limited to corticosteroid-refractory patients, intensive LCAP was associated with favorable long-term outcomes. In our previous postmarketing study, intensive LCAP was identified as the only factor that was significantly related to remission after LCAP [15]. Therefore, these results indicate that intensive LCAP is favorable not only for remission induction but also for long-term outcomes in corticosteroid-refractory UC patients.

In a study investigating the long-term outcomes of CAP therapy in 90 patients, Takayama et al. [24] reported that concomitant thiopurine maintenance therapy significantly reduced the rate of hospitalization. The results of the present study also suggested that concomitant thiopurine maintenance therapy was associated with favorable long-term outcomes of corticosteroid non-refractory patients. However, our data demonstrated that the dose of 5-ASA and use of concomitant thiopurine maintenance therapy did not affect outcomes after LCAP overall. In the present, retrospective, uncontrolled, observational study, physicians were allowed to alter the treatment regimen during the observation period on an individual basis; therefore, it is possible that the results were biased by the treatments during the observation period. Hence, a well-designed prospective randomized controlled trial is warranted to clarify an appropriate maintenance treatment after induction of remission with LCAP.

Takayama et al. [24] also reported that the efficacy of CAP was relatively high when CAP was re-introduced in 20 patients who achieved remission with prior CAP, with a response rate or 80% (16 of 20), but was ineffective in those who failed to respond to prior CAP [response rate, 40.0% (2 of 5)]. The results of the present study demonstrated a high response rate (>85%) by re-treatment with LCAP for patients who relapsed during the observation period. Therefore, these results suggest that favorable outcomes can be expected when the patient is treated with LCAP repeatedly for relapse if prior LCAP treatment was effective.

This study had several limitations. First, relapse was defined by the addition of treatments that are normally used for moderate-to-severe active UC, such as systemic corticosteroid, TAC, and/or anti-TNFα administration. Therefore, patients who were treated with topical treatment were not considered as relapsed. Second, the efficacy of re-induction treatments for relapsed cases was retrospectively assessed based on a chart review, but no objective definition was determined prospectively.

In conclusion, this study provides large-scale, real-world data on the long-term outcomes of treatment with LCAP for moderate-to-severe UC. The findings of this study have shown that the majority of patients (>60%) with UC treated with LCAP achieved clinical remission within 1 year without relapse. A higher CAI and leukocyte count following LCAP were risk factors for relapse of UC. Intensive LCAP was associated with favorable long-term outcomes for corticosteroid-refractory patients. Furthermore, re-treatment with LCAP is effective for relapse in patients who responded to LCAP prior to induction of remission.

## Electronic supplementary material

Below is the link to the electronic supplementary material.

Supplementary material 1 (DOC 73 kb)
