# Supplementary material for: A multicenter, retrospective, observational study of the clinical outcomes and risk factors for relapse of ulcerative colitis at 1 year after leukocytapheresis
Source: J Gastroenterol. 2017 Jun 8;53(3):387–96. doi: 10.1007/s00535-017-1356-8 (PMC5847179; doi:10.1007/s00535-017-1356-8)
Supplement: Supplementary file 1 — Supplementary material 1 (DOC 73 kb) [file 535_2017_1356_MOESM1_ESM.doc]

S1. Candidate factors for relapse by univariate analysis

| Item | Factor | No. of patients | 1-year cumulative relapse-free rate | *p-value* |
| --- | --- | --- | --- | --- |
| Before LCAP | | | | |
| Age, years | <65  ≥65 | 264  36 | 63.9%  61.5% | 0.926^a^ |
| Weight, kg | ≤50  <50–60  <60–70  >70 | 82  87  68  39 | 68.5%  63.3%  56.8%  62.4% | 0.522^a^ |
| Sex | Male  Female | 184  116 | 62.4%  65.6% | 0.538^a^ |
| Disease duration, years | <1  ≤1–3  ≤3–5  ≤5–10  ≥10 | 66  46  42  49  90 | 62.4%  68.6%  66.5%  57.0%  61.5% | 0.817^a^ |
| Lichtiger CAI | 5–6  7–11  ≥12 | 39  159  102 | 60.0%  66.6%  60.5% | 0.580^a^ |
| Disease extent | Total  Left-sided  Others | 171  110  18 | 63.2%  63.7%  71.4% | 0.789^a^ |
| Response to corticosteroid | Refractory  Nonrefractory | 193  105 | 60.5%  70.5% | 0.049^a^ |
| Previous use of corticosteroid | Yes  No | 226  73 | 62.6%  66.3% | 0.581^a^ |
| Laboratory data |  |  |  |  |
| Leukocyte count, /mm^3^ | Continuous value (1000 U) | 290 | － | 0.703^b^ |
| Erythrocyte count, ×10^4^/mm^3^ | Continuous value (100 U) | 290 | － | 0.972^b^ |
| Platelet count, ×10^4^/mm^3^ | Continuous value (10 U) | 290 | － | 0.241^b^ |
| Hemoglobin level, g/dl | Continuous value (1 U) | 290 | － | 0.727^b^ |
| CRP level, mg/dl | Continuous value (1 U) | 290 | － | 0.526^b^ |
| Erythrocyte sedimentation rate, mm/h | Continuous value (10 U) | 155 | － | 0.319^b^ |
|  |  |  |  |  |
| After LCAP | | | | |
| Lichtiger CAI | 0  1,2  3,4 | 61  152  87 | 69.9%  69.8%  48.0% | <0.001^a^ |
| EI | 0  1  2 | 35  43  28 | 67.6%  66.7%  46.4% | 0.042^a^ |
| Laboratory data |  |  |  |  |
| Leukocyte count, /mm^3^ | Continuous value (1000 U) | 265 | － | <0.001^b^ |
| Erythrocyte count, ×10^4^/mm^3^ | Continuous value (100 U) | 264 | － | 0.914^b^ |
| Platelet count, ×10^4^/mm^3^ | Continuous value (10 U) | 264 | － | 0.898^b^ |
| Hemoglobin level, g/dl | Continuous value (1 U) | 264 | － | 0.954^b^ |
| CRP level, mg/dl | Continuous value (1 U) | 263 | － | 0.835^b^ |
| Erythrocyte sedimentation rate, mm/h | Continuous value (10 U) | 138 | － | 0.789^b^ |
|  |  |  |  |  |
| Concomitant medications | | | | |
| During LCAP | | | | |
| 5-ASA | Yes  No | 286  14 | 63.2%  71.4% | 0.633^a^ |
| Thiopurine | Yes  No | 101  199 | 64.8%  63.0% | 0.933^a^ |
| Corticosteroid | Yes  No | 183  117 | 60.2%  69.0% | 0.118^a^ |
| Beginning of the observation period | | | | |
| Corticosteroid | Yes  No | 163  137 | 58.6%  69.6% | 0.034^a^ |
| During of the observation period | | | | |
| 5-ASA | Yes  No | 280  20 | 64.1%  57.9% | 0.486^a^ |
| Thiopurine | Yes  No | 130  170 | 65.3%  62.3% | 0.670^a^ |
|  |  |  |  |  |
| LCAP treatment status |  |  |  |  |
| Number of sessions | 5-7  8-9  10 | 46  17  228 | 66.7%  63.7%  62.0% | 0.767 ^a^ |
| Frequency of LCAP | Weekly  Intensive | 88  203 | 57.0%  65.4% | 0.186 ^a^ |

5-ASA, 5-aminosalicylic acid; CAI, clinical activity index; CRP, C-reactive protein; EI, endoscopic index; LCAP, leukocytapheresis.

^a^Calculated using the log-lank test.

^b^Calculated using the Cox proportional hazard model.
